# Corneal Epithelial Thickness Maps in Eyes with Mild and Moderate Keratoconus

**DOI:** 10.3390/jcm14041256

**Published:** 2025-02-14

**Authors:** Patryk Mlyniuk, Magdalena Kaszuba-Modrzejewska, Jagoda Rzeszewska-Zamiara, Ilona Piotrowiak-Slupska, Bartlomiej J. Kaluzny

**Affiliations:** 1 Department of Ophthalmology, Collegium Medicum, Nicolaus Copernicus University, ul. Ujejskiego 75, 85-168 Bydgoszcz, Poland; mkm@cm.umk.pl (M.K.-M.); j.rzeszewska@cm.umk.pl (J.R.-Z.); ilona.piotrowiak@cm.umk.pl (I.P.-S.); b.kaluzny@cm.umk.pl (B.J.K.); 2 Oftalmika Eye Hospital, ul. Modrzewiowa 15, 85-631 Bydgoszcz, Poland

**Keywords:** corneal epithelial thickness, keratoconus, optical coherence tomography

## Abstract

**Background/Objectives:** The evaluation of the differences in corneal epithelial thickness profiles in healthy eyes and eyes with mild and moderate stages of keratoconus, using optical coherence tomography (OCT). **Methods:** Fifty-two healthy eyes (group 0), forty-one eyes with mild keratoconus (group I), and thirty eyes with moderate keratoconus (group II) were included in this study. Only one of the patient’s eyes was enrolled, and they were divided into groups using the Amsler–Krumeich (A–K) classification—stage I and II. All patients underwent a visual acuity assessment, slit-lamp examination, corneal tomography, and automatic mapping of corneal thickness and epithelial thickness on a diameter of 9 mm. Corneal tomography with a Placido/Scheimpflug instrument (Sirius, CSO, Florence, Italy) and OCT with a corneal adaptor module (Avanti RTVue XR, Optovue, Lombard, IL, USA) were used. **Results:** Minimum corneal epithelium thickness was 49.5, 43, and 40 µm in groups 0, I, and II, respectively (Kruskal–Wallis test, *p* < 0.001). A moderate correlation was found between minimum epithelial thickness and the apex curvature (Pearsons’s coefficient r = −0.62, *p* < 0.001) and posterior radius of central corneal curvature (Pearsons’s coefficient r = 0.62, *p* < 0.001). The difference between minimum and maximum epithelial thickness showed a high correlation (r = −0.770, *p* < 0.001). In groups I and II, on corneal epithelial thickness maps the thinnest sector, located inferiorly and temporally to the center, was surrounded by sectors with increased thickness. **Conclusions:** At the apex of the cone, the corneal epithelium becomes thinner, and a thicker ring forms around the cone. Although there is a moderate-to-strong correlation to parameters linked with the severity of keratoconus, and minimum epithelial thickness as well as the minimum–maximum difference, it is not possible to establish cut-off values for stages I and II in the Amsler–Krumeich (A–K) classification.

## 1. Introduction

The cornea is the transparent, outermost part of the anterior segment of the eye. Along with the tear film and intraocular lens, it plays an important role in focusing light rays on the retina, and also in the perception of visual stimuli [1,2,3,4,5]. Additionally, along with the tear film, it acts as a mechanical barrier against the factors of the external environment, including variations in atmospheric pressure, eyelid movements, eye rubbing, etc. [1,2,3,4,5,6,7].

The cornea externally consists of epithelium, anterior limiting lamina (Bowman’s membrane), stroma, Dua’s layer, posterior limiting lamina (Descemet’s membrane), and endothelium [1,2,3,4,5]. In turn, the epithelium is composed uniformly of 5–7 layers of nonkeratinized stratified squamous cells and its thickness is 45 to 60 µm [1,2,3,4]. The corneal epithelium prevents the entrance of foreign bodies into the deeper layers of the cornea while allowing the transport of oxygen and nutrients [1,2,3,4]. Moreover, due to its ability to multiply quickly, it compensates for irregularities of the stroma, ensuring a smooth optical surface [1,2,3,4].

As a result of ectatic corneal diseases (ECDs), morphometric changes of the cornea occur, such as corneal thinning, increased steepness, and irregular astigmatism [8,9,10,11,12,13,14,15]. In addition, the stiffness of the cornea is reduced, which directly affects its biomechanical properties, i.e., elasticity, tensile strength, and the ability to distribute stresses [2,3,5,6,7,16]. The main ECDs include keratoconus (KC), pellucid marginal degeneration (PMD), and keratoglobus [8,9,10,11,12,13,14,15].

The most common ECD is keratoconus, which is a progressive, bilateral disorder which may be associated with ocular inflammation. The central or paracentral part of the cornea gradually becomes thinner and finally develops a cone-shaped protrusion. The early diagnosis of keratoconus is of great importance for refractive surgery to prevent postoperative ectasia and to improve postoperative healing and prognosis [8,11,12,13]. The detection of the early stage of keratoconus is possible by more advanced techniques, such as corneal tomography due to its ability to generate three-dimensional images of the anterior and posterior corneal elevation even in subclinical disease [12,13,17].

It has been hypothesized that changes in the corneal epithelium develop in the subclinical stage of keratoconus. This layer can alter and rebuild itself in response to irregularities and changes in the stroma caused by KC [9,10,11,18]. Reinstein et al. [18] introduced these types of compensatory changes of the epithelial layers as a “doughnut pattern”, thinner over the cone and thicker in the surrounding area, which has been confirmed in many studies [9,10,11].

Currently, there are several grading scales for keratoconus, including the Amsler–Krumeich (A–K) classification [19,20], ABCD Grading System [21], and Sandali classification [22,23], which can be used to classify keratoconus according to its severity, taking into account parameters such as corneal topography, corneal thickness, refraction, best corrected visual acuity (BCVA), biomicroscopy, and parameters obtained with anterior segment optical coherence tomography (AS-OCT) [19,20,21,22,23]. The A–K classification and ABCD Grading System include similar parameters for assessing the severity of keratoconus and are useful for the identification and long-term monitoring of patients with mild keratoconus. On the other hand, the Sandali classification evaluates structural changes in the cornea, so it is not interchangeable with the above. It is indicated in the classification and monitoring of keratoconus in advanced stages [19,20,21,22,23]. However, none of these scales take into account the thickness of the corneal epithelium, which undergoes significant changes and potentially could be used to classify the severity of the disease more precisely. Some studies analyze the corneal epithelial thickness, but the majority of them compare healthy eyes with keratoconus, without distinguishing between disease stages, or sub-clinical keratoconus [24,25,26,27,28].

The aim of this study is to evaluate the differences in the corneal epithelial thickness profiles on a diameter of 9 mm in healthy eyes and eyes with mild and moderate stages of keratoconus, using high-resolution optical coherence tomography in the Fourier domain (Avanti RTVue XR, Optovue, Lombard, IL, USA) with a corneal adaptor module.

## 2. Methods

### 2.1. Study Design

This prospective study was conducted at the Department of Ophthalmology, Collegium Medicum in Bydgoszcz Nicolaus Copernicus University in Torun (Poland) and Oftalmika Eye Hospital in Bydgoszcz. The principles of the Declaration of Helsinki, the International Conference on Harmonization Good Clinical Practice guidelines, and all applicable laws and regulations were applied. The study was approved by the Ethics Committee on Clinical Investigation of Nicolaus Copernicus University.

### 2.2. Participants

Three groups of patients participated in the study: group 0 consisted of 52 healthy eyes, group I consisted of 41 eyes with mild keratoconus, and group II consisted of 30 eyes with moderate keratoconus. Only one of the patient’s eyes was enrolled. The eyes were divided into groups using the Amsler–Krumeich (A–K) classification—stage I and II.

### 2.3. Study Protocol

All patients underwent a refraction examination, slit-lamp biomicroscopy, and corneal tomography with a Placido/Scheimpflug instrument (Sirius, CSO, Florence, Italy). Using a Sirius device, the horizontal visible iris diameter (HVID), average keratometry, keratometry cylinder, the anterior and posterior radius of central corneal curvature (3 mm), corneal apex curvature, and anterior chamber depth were assessed.

**Figure 1:**
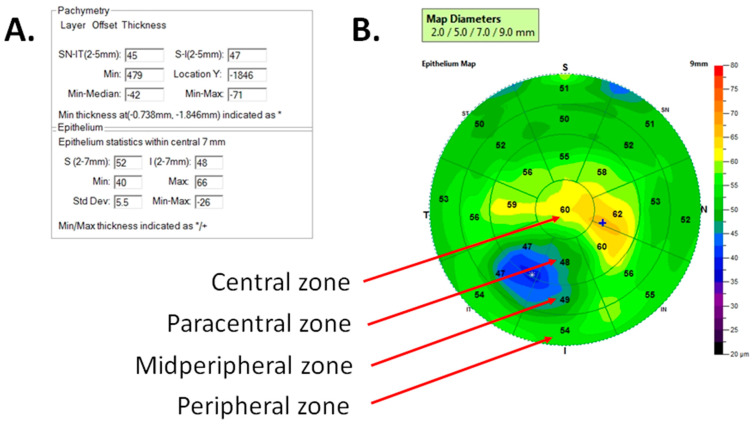
Example result of anterior segment optical coherence tomography (OCT) measurement of right eye with keratoconus. (**A**) Pachymetric data and epithelium statistics within central 7 mm. (**B**) Corneal epithelial thickness map with its zones. * means min thickness of the epithelium and + means max thickness of the epithelium.

Corneal thickness and epithelial thickness mapping were performed using a high-resolution Fourier-domain optical coherence tomography device (Avanti RTVue XR, Optovue, USA) with a corneal adaptor module. This device measured the thickness of the cornea and its epithelium on a diameter of 9 mm. The automatic segmentation mode was used, which provides reliable data in fewer irregular corneas only, thus the eyes with advanced keratoconus were not enrolled in the study. The obtained maps allow the assessment of 4 zones of the cornea: central 2 mm, paracentral 2 to 5 mm, mid-peripheral 5 to 7 mm, and peripheral 7 to 9 mm. Additionally, each zone (without central) was divided into eight sectors, Figure 1 [29]. Epithelial statistics within the central 7 mm were also used for analysis, such as epithelial thickness in the inferior and superior corneal hemispheres (2–7 mm). The minimum and maximum thickness of the corneal epithelium enabled a qualitative assessment of the distribution of the corneal epithelium.

### 2.4. Statistical Analysis

The statistical analysis was performed using Statistica 13.3 (TIBCO Software Inc. Santa Clara, CA, USA). The statistical results of the parameters for normally distributed continuous variables are presented as the mean and standard deviation (SD), or as the median and interquartile range (Q1, Q3) for non-normally distributed variables. The Shapiro–Wilk test was used to assess the normality of the distribution. In the case of the normal distribution, the variability was assessed using the ANOVA test, and for the non-normal distribution, the differences were tested using the Kruskal–Wallis test. To assess the differences between respective groups, the Student’s *t*-test was used for normally distributed data and the Mann-Whitney-Wilcoxon as a post hoc test for non-normally distributed data. Pearson’s correlation coefficient (r) was calculated to investigate the dependencies between selected continuous variables. The *p*-value < 0.05 was considered as statistically significant.

## 3. Results

The basic morphometric parameters of the cornea were assessed. The simulated keratometry increased with the advancement of the keratoconus stage and the median was 42.95 D in healthy eyes, 44.34 D in eyes with mild keratoconus, and 47.8 D in eyes with moderate keratoconus (Kruskal–Wallis test, *p* < 0.001). The baseline characteristics of the enrolled eyes are presented in Table 1.

Table 2 provides the parameters of the central cornea of 3 mm diameter assessed with the Placido/Scheimpflug instrument in groups. In eyes with moderate keratoconus, the corneal apex curvature was the highest (Kruskal–Wallis test, *p* < 0.001). As the severity of keratoconus increases, both the anterior and posterior radius of the central corneal curvature decreases (Kruskal–Wallis test, *p* < 0.001).

Table 3 presents the results of the measurements of the thickness of the cornea and epithelium in groups. The central (ANOVA, *p* < 0.001) and minimum corneal thickness (Kruskal–Wallis test, *p* < 0.001) decreased with the advance of keratoconus, whereas central epithelial thickness did not show a statistical difference between I and II. The epithelial focal thinning was calculated as the difference between the minimum and maximum thickness of the corneal epithelium (MIN–MAX). As the keratoconus progressed, more negative MIN–MAX values were observed (Kruskal–Wallis test, *p* < 0.001).

**Figure 2:**
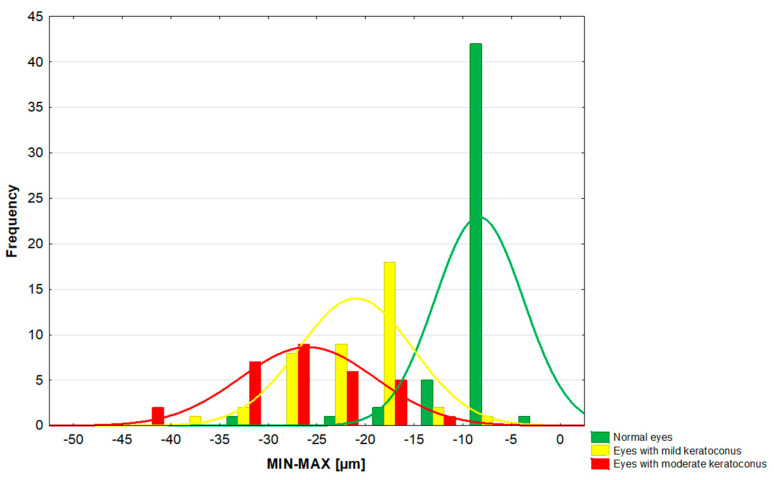
Histogram of the focal thinning of the corneal epithelium (MIN–MAX).

Based on the obtained MIN–MAX results, the ranges of values of this parameter were calculated. Healthy eyes and eyes with early and moderate keratoconus were −8.0 to −4.0, −13.0 to −11.0, and −43.0 to −33.0 µm, respectively. Moreover, a histogram of the MIN–MAX values in groups was produced (Figure 2). Distribution curves for groups overlap, especially for groups I and II.

The correlations between the parameters of the corneal curvature and the central, minimum, maximum, MIN–MAX difference, and superior and inferior thickness of the corneal epithelium were also calculated. A moderate negative correlation was found between minimum epithelial thickness and several parameters that express the severity of keratoconus, including the apex curvature (Pearsons’s coefficient r = −0.62, *p* < 0.001). The difference between minimum and maximum epithelial thickness showed a high correlation (r = −0.770, *p* < 0.001). As the corneal apex curvature increases, the minimum epithelial thickness decreases, and its maximum thickness increases. In contrast, there is no or weak correlation between the parameters of the corneal curvature and epithelial thickness in the superior and inferior corneal hemispheres (*p* < 0.05). The detailed results are presented in Table 4.

**Figure 3:**
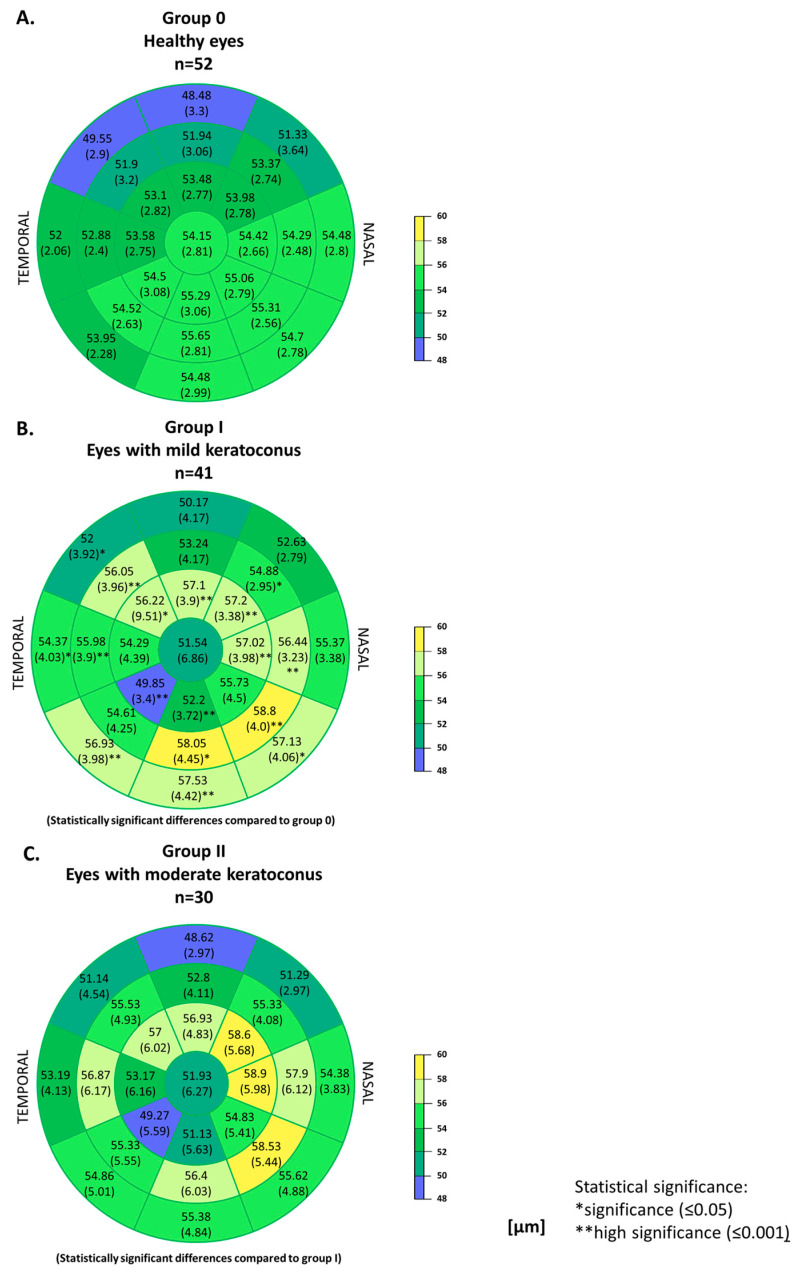
Corneal epithelial thickness maps. (**A**) in healthy eyes, (**B**) in eyes with mild keratoconus and statistical differences to healthy eyes, and (**C**) in eyes with moderate keratoconus and statistical differences to mild keratoconus; mean values.

The thickness maps of corneal epithelium and differences between healthy eyes and eyes with mild to moderate keratoconus were assessed. Figure 3 presents maps with mean values for all three groups. In healthy eyes, the corneal epithelium was thicker in the inferior and inferior-nasal sectors of the cornea. On the other hand, in the superior and superior-temporal sectors of the cornea, the epithelium was thinner. As it moves away from the center of the cornea, the thickness of its epithelium decreases except for the nasal, inferior-nasal, inferior, and inferior-temporal sectors in the paracentral zone of the cornea. In the case of eyes with mild keratoconus, significant changes in the thickness of the corneal epithelium were observed in individual sectors compared to healthy eyes. The corneal epithelium was significantly thinned in the central zone of the cornea and in the inferior-temporal and inferior sectors of the paracentral zone of the cornea (*T*-test, *p* < 0.05). Its thickness increased in the superior, superior-temporal, superior-nasal, and nasal sectors of the paracentral zone of the cornea (*T*-test, *p* < 0.05). In the mid-peripheral zone of the cornea, a thicker epithelium compared to healthy eyes was observed in the inferior, inferior-nasal, nasal, temporal, superior-nasal, and superior-temporal sectors of the cornea (*T*-test, *p* < 0.05). In the case of the peripheral zone of the cornea, an increase in the thickness of the epithelium was observed in the inferior, inferior-nasal, inferior-temporal, temporal, and superior-temporal sectors of the cornea (*T*-test, *p* < 0.05). On the other hand, in eyes with moderate keratoconus compared to mild keratoconus, no differences in corneal epithelial thickness were observed in any of the sectors (*T*-test, *p* > 0.05).

## 4. Discussion

The importance of assessing the thickness of the corneal epithelium in the eyes of candidates for refractive surgery has increased significantly in recent years. It enables not only the exclusion of eyes with a diagnosis of keratoconus, but also the identification of patients at increased risk of iatrogenic corneal ectasia after laser vision correction [30]. In this study, the thickness profile of the corneal epithelium was analyzed in eyes with mild to moderate keratoconus. The diagnosis of keratoconus at an advanced stage is usually not difficult; therefore, only patients with the initial stages of the disease were enrolled in this study, as they cause the most diagnostic uncertainty. 

Patients were divided into groups according to the Amsler–Krumeich (A–K) classification based on the analysis of corneal topography, corneal thickness, refraction, and biomicroscopy. Despite the limitations of the A–K classification, it is still the most commonly used. It is also reported to be more accurate in identifying and monitoring mild forms of keratoconus compared to the Sandali classification. Sandali classification, based on the parameters obtained with AS-OCT, seems to be more appropriate for eyes with more advanced stages of keratoconus, in which it is important to select an appropriate treatment method based on structural changes in the cornea [23].

The obtained significant differences between the groups in the parameters of keratometry and central corneal thickness, on the one hand, result from the A–K classification. On the other hand, they confirm that the analyzed groups differ significantly in terms of the stage of the disease. The intention was to determine what changes occur in the pachymetric maps of the corneal epithelium with the increasing severity of keratoconus. In previous studies, based on the Fourier-domain OCT, a mean central corneal thickness of 50.45 to 53.4 µm was obtained [26,27,28,31]. Although many studies describe the thinning of the corneal epithelium in the center of the cornea with keratoconus compared to normal eyes [24,25,28,32], it is believed that this is not pathognomonic for keratoconus, but only suggestive for the diagnosis of the disease [26]. In our study, the central corneal epithelium thickness in the control group was 54 µm, and it was 52 µm in mild and 50 µm in moderate keratoconus (Kruskal–Wallis test, *p* = 0.02). Rocha et al. reported a central corneal epithelial thickness of 50.45 µm in healthy eyes and 41.18 µm in eyes with keratoconus. The difference was significant [26]. Unlike us, they did not divide patients into groups depending on the severity of the disease. Li et al. observed no significant differences in the thickness of the central epithelium in eyes with keratoconus; however, they observed significantly thinner corneal epithelium inferiorly, lower minimum epithelial thickness, greater S-I, and a more negative MIN–MAX compared to normal eyes [27]. We found a significant difference between the group of healthy eyes and the group of eyes with mild keratoconus in terms of superior, central, minimum, and maximum thickness of the corneal epithelium, and a non-significant difference in the inferior sector. The groups of mild and moderate keratoconus differed significantly only in the inferior hemisphere of the cornea (Figure 2). The difference between our results and those obtained by Li et al. may result from grouping based on the severity of keratoconus. Li et al. considered only eyes with keratoconus and normal eyes, while we divided the keratoconus eyes into groups according to the severity of the disease.

A decrease in the central corneal epithelial thickness in form fruste keratoconus has been reported by Temstet et al. [28]. The Optovue RTVue device was used for these measurements. A sensitivity of 88.9% (95% confidence interval [CI], 73.9−96.1%) and a specificity of 59.5% (95% CI, 44.5–72.9%) was demonstrated for the 52 µm cutoff for central epithelial thickness. Moreover, the point of the minimum thickness of the corneal epithelium was shown to correspond to the point of minimum thickness of the cornea and was found in the inferotemporal and inferonasal sectors for the form fruste and moderate keratoconus and in the superotemporal and superonasal sectors for healthy eyes [28]. A significant decrease in the mean epithelial thickness in the thinnest corneal zone with the advancement of keratoconus was reported [28]. Furthermore, they found no other statistically significant differences between the form fruste keratoconus group and the control group except for the minimum corneal thickness, which was lower in the form fruste group. In this study, the minimum epithelial thickness was also observed in these quadrants; moreover, statistically significant differences in the thickness of the corneal epithelium between the mild and moderate keratoconus eye groups were significant only in the inferotemporal and peripheral inferior sectors. Xu et al. find no significant differences in inferior epithelium thickness in sub-clinical keratoconus eyes. However, significant thinning of the corneal epithelium in the central region of eyes with sub-clinical keratoconus and with keratoconus has been reported [33].

In this study, the thickness of the epithelium in the nasal quadrants was higher than in the temporal quadrants in all groups, which also coincides with the results of most other studies [27]. Reinstein et al. [24] suggest that such a pattern of the thickness map of the corneal epithelium in healthy eyes may be due to the greater pressure of the eyelids on the temporal cornea compared to the nasal one. In addition, the difference between the nasal and temporal thickness of the corneal epithelium increased with the severity of keratoconus. This, in turn, may result from the characteristic “doughnut pattern” and from the fact that its diameter increases with the advancement of keratoconus [34].

We observed significant differences in the minimum thickness of the corneal epithelium and the MIN–MAX value in eyes with mild keratoconus compared to eyes with moderate keratoconus. Moreover, they were moderately or strongly correlated with parameters linked with the severity of keratoconus, like corneal apex curvature or the posterior radius of central corneal curvature. The MIN–MAX thickness difference seems to be the most promising parameter in keratoconus grading. Unfortunately, the distribution curves of MIN–MAX thickness difference between normal, stage I, and stage II keratoconus show large areas of overlap (Figure 2). Thus, it is not possible to set cut-off values for these clinical conditions.

In this study, 9 mm diameter maps were analyzed, making the data more comprehensive compared to the previous studies in which 6 mm corneal epithelial thickness maps were analyzed. Since keratoconus is usually located in the inferior temporal quadrant—the center of corneal epithelial thinning is also decentralized [28]. The diameter of the thinning and the ring around it depends on the severity of the keratoconus [34]. Therefore, a map of the epithelium with a diameter of 6 mm can only include a fragment of the ring forming the characteristic “doughnut pattern”. Although it is believed that a map of this diameter may be sufficient for diagnosis, because the apex of the keratoconus is usually inside the central area of the cornea with a diameter of 5 mm, a map with a larger area produces a more complete picture and also allows the assessment of deviations in pathologies located more peripherally, like pellucid marginal degeneration [35].

In our study, a pachymetric analysis of corneas with advanced keratoconus was not performed, which might be considered as a limitation of the study. These eyes were excluded because mapping the corneal epithelium in eyes with highly irregular corneas, especially with signs of scarring, does not provide reliable and repeatable results in automatic segmentation mode and sometimes it is even troublesome in manual mode. Moreover, it should be remembered that the data for individual sectors were the average calculated by the device from a number of measurement points in a given area. There is no information about the deviation of individual measurements from the mean. This deviation may be greater in the more advanced stages of the disease, which may not be seen in the average values.

## 5. Conclusions

The analysis of corneal epithelial thickness maps may prove to be a significant supplement to the current diagnosis of keratoconus, as well as the assessment of disease progression. In addition, from a clinical point of view, it is important to know the epithelial thickness profile at different stages of keratoconus and to complete the grading scales with these parameters. In the case of eyes with keratoconus, the characteristic “doughnut pattern” is formed in the map of the corneal epithelium in its inferior-temporal sectors. At the apex of the cone, the corneal epithelium becomes thinner, and a thicker ring forms around the cone. Although there is a moderate-to-strong correlation of parameters linked with the severity of keratoconus and minimum epithelial thickness as well as the MIN–MAX difference, it is not possible to set cut-off values for stages I and II in the Amsler–Krumeich (A–K) classification.

## Figures and Tables

**Table 1:** Baseline characteristics of the enrolled eyes.

	Group 0— Healthy Eyes	Group I— Eyes with Mild Keratoconus	Group II— Eyes with Moderate Keratoconus	ANOVA/ Kruskal– Wallis Test **	*p* (Group 0–Group I)	*p* (Group I–Group II)	*p* (Group 0–Group II)
	n = 52	n = 41	n = 30				
Age [years]	31.31 (5.53)	30.37 (7.7)	30.87 (8.35)	0.68			
Spherical equivalent [D]	−2.26 (3.58)	−1.13 (1.38)	−4.16 (3.56)	**<0.001**	0.05	**<0.001**	0.05
Refractive astigmatism [D]	−0.5 (−0.75–0.0) *	−1.25 (−2.5–0.0) *	−2.5 (−3.0–−1.5) *	<0.001 **	**0.02**	**0.004**	**<0.001**
Mean keratometry [D]	42.95 (42.24–43.96) *	44.34 (43.72–46.18) *	47.8 (46.16–50.83) *	<0.001 **	**<0.001**	**<0.001**	**<0.001**
Keratometry cylinder [D]	−0.9 (−1.38–−0.57) *	−2.54 (−3.41–−1.71) *	−3.51 (−4.59–−1.94) *	<0.001 **	**<0.001**	0.09	**<0.001**
Horizontal visible iris diameter [mm]	12.05 (11.96–12.34) *	12.39 (12.16–12.7) *	12.41 (11.98–12.57) *	<0.001 **	**<0.001**	0.22	0.11
Anterior chamber depth [mm]	3.64 (0.32)	3.75 (0.32)	3.82 (0.39)	**<0.05**	0.17	0.45	0.1

* Median (range). ** Kruskal–Wallis test.

**Table 2:** Parameters of the central cornea in groups; mean values (SD).

	Group 0— Healthy Eyes	Group I— Eyes with Mild Keratoconus	Group II— Eyes with Moderate Keratoconus	ANOVA/ Kruskal- Wallis Test **	*p* (Group 0–Group I)	*p* (Group I–Group II)	*p* (Group 0–Group II)
	n = 52	n = 41	n = 30				
Corneal apex curvature [D]	44.46 (43.48–45.37) *	54.91 (51.31–57.88) *	58.83 (55.4–61.4) *	<0.001 **	**<0.001**	**<0.001**	**<0.001**
Anterior radius of central corneal curvature [mm]	7.86 (7.66–7.98) *	7.48 (7.21–7.78) *	6.92 (6.34–7.22) *	<0.001 **	**<0.001**	**<0.001**	**<0.001**
Posterior radius of central corneal curvature [mm]	6.43 (6.31–6.62) *	6.12 (5.52–6.72) *	5.02 (4.64–5.47) *	<0.001 **	**0.01**	**<0.001**	**<0.001**

* Median (range). ** Kruskal–Wallis test.

**Table 3:** The thickness of the cornea and corneal epithelium; mean values (SD).

	Group 0— Healthy Eyes	Group I— Eyes with Mild Keratoconus	Group II— Eyes with Moderate Keratoconus	ANOVA/ Kruskal– Wallis Test **	*p* (Group 0–Group I)	*p* (Group I–Group II)	*p* (Group 0–Group II)
	n = 52	n = 41	n = 30				
Central corneal thickness [µm]	537.1 (27.00)	499.59 (38.86)	446.47 (29.84)	**<0.001**	**<0.001**	**<0.001**	**<0.001**
Minimum corneal thickness [µm]	530 (508.5–549.8) *	461 (446–479.75) *	403 (386–423.75) *	<0.001 **	**<0.001**	**<0.001**	**<0.001**
Central epithelial thickness [µm]	54 (52–56.25) *	52 (48–56) *	50 (47–56) *	0.02 **	0.06	0.82	0.06
Minimum epithelial thickness [µm]	49.5 (47.75–51) *	43 (40–46.25) *	40 (37.25–44.5) *	<0.001 **	**<0.001**	**0.03**	**<0.001**
Maximum epithelial thickness [µm]	57.5 (56–60) *	64 (61–66) *	65.5 (62.25–70) *	<0.001 **	**<0.001**	0.09	**<0.001**
MIN–MAX [µm]	−8 (−9 to −6) *	−19 (−25 to −17) *	−26 (−30 to −20.25) *	<0.001 **	**<0.001**	**0.003**	**<0.001**
Superior epithelial thickness [µm]	53.02 (3.14)	55.63 (4.19)	55.2 (4.78)	**0.004**	**0.004**	0.69	0.06
Inferior epithelial thickness [µm]	56 (53.75–57.25) *	55 (52–57) *	52 (50–55.75) *	0.003 **	0.14	0.09	**0.003**

* Median (range). ** Kruskal–Wallis test.

**Table 4:** Pearson’s correlation coefficient r of the parameters of the corneal curvature with the central, minimum, maximum, and superior and inferior thickness of the corneal epithelium.

	Corneal Epithelial Thickness											
	Central		Minimum		Maximum		MIN–MAX		Superior		Inferior	
	r	*p*	r	*p*	r	*p*	r	*p*	r	*p*	r	*p*
Keratometry average [D]	−0.25	**<0.001**	−0.4	0.94	0.34	**<0.001**	−0.46	**<0.001**	0.13	**<0.001**	−0.12	**<0.001**
Corneal apex curvature [D]	−0.14	0.21	−0.62	**<0.001**	0.62	**<0.001**	−0.77	**<0.001**	0.26	**<0.001**	−0.29	**<0.001**
Anterior radius of central corneal curvature [mm]	0.27	**<0.001**	0.39	**<0.001**	−0.35	**<0.001**	0.47	**<0.007**	−0.16	**<0.001**	0.14	**<0.001**
Posterior radius of central corneal curvature [mm]	0.3	**<0.001**	0.62	**<0.001**	−0.32	**<0.001**	0.45	**<0.001**	−0.13	**<0.001**	0.16	**<0.001**

## Data Availability

https://doi.org/10.18150/TZOMOG.
